# PyFCG: A Python toolkit for constructionist language processing and learning

**DOI:** 10.1016/j.isci.2026.118129

**Published:** 2026-11-05

**Authors:** Paul Van Eecke, Katrien Beuls

**Affiliations:** 1 Artificial Intelligence Laboratory, Vrije Universiteit Brussel, Pleinlaan 2, 1050 Brussels, Belgium; 2 Namur Digital Institute, Université de Namur, 61 rue de Bruxelles, 5000 Namur, Belgium

**Keywords:** construction grammar, scientific software, Fluid Construction Grammar, emergent communication

## Abstract

This article introduces PyFCG, an open-source scientific software toolkit that enables its users to efficiently integrate state-of-the-art constructionist language processing and learning into larger Python projects. The toolkit provides a fully Pythonic interface to the leading computational construction grammar framework, Fluid Construction Grammar (FCG), facilitating its swift integration with Python’s rich ecosystem of libraries, in particular those implementing today’s most popular machine learning techniques. Apart from a general description of the toolkit, this article provides three illustrative case studies that demonstrate how PyFCG facilitates (i) the formalization and computational operationalization of construction grammar analyses, (ii) the learning of usage-based construction grammars from corpora of language use, and (iii) the implementation of multi-agent emergent communication experiments that combine symbolic and neural computation.

## Graphical abstract

**Figure:**
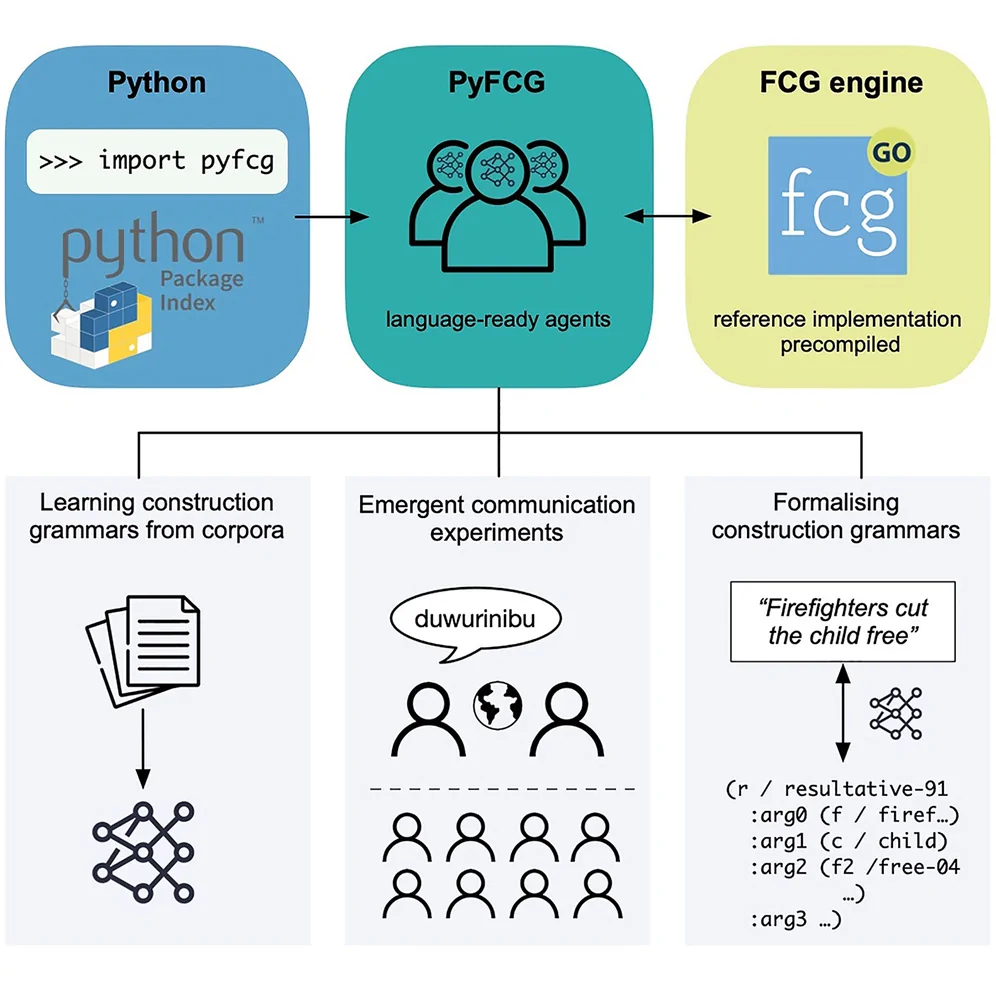

## Teaser

Computer science; Software engineering; Software

## Introduction

Fluid Construction Grammar (FCG)^1^^,^^2^ is a computational construction grammar framework that provides a collection of high-level building blocks for representing, processing, and learning fully operational construction grammars. The FCG framework is conceived as an open instrument that is not tied to a particular construction grammar theory but that strives for compatibility with any linguistic theory that adheres to the most fundamental tenets underlying constructionist approaches to language.^3^^,^^4^^,^^5^ As such, it subscribes to the view (i) that language users dynamically build up their own linguistic systems as they communicate with other members of their community, (ii) that these linguistic systems can be captured as a network of form-meaning mappings called constructions, and (iii) that these constructions can pair forms and meanings of arbitrary complexity and degree of abstraction, thereby facilitating a uniform handling of both compositional and non-compositional linguistic phenomena.^6^

Fluid Construction Grammar is primarily being used as a framework for modeling the language representation, processing, and learning components in agent-based models of linguistic communication. Such models simulate the emergence, evolution, and acquisition of natural languages in populations of artificial agents that take part in situated communicative interactions modeled after those that human language users continuously engage in.^7^^,^^8^ Other common uses of FCG include the formalization and computational operationalization of construction grammar analyses^9^^,^^10^ and the corroboration of construction grammar theories with empirical data.^11^

This article introduces PyFCG as an open-source scientific software toolkit for integrating state-of-the-art constructionist language processing and learning into Python projects. The toolkit provides a fully Pythonic interface to FCG, thereby facilitating its seamless integration with Python’s rich ecosystem of libraries. Apart from a general description of the toolkit, we present three illustrative case studies that demonstrate how PyFCG can be used for formalizing and computationally operationalizing construction grammar analyses, for learning usage-based construction grammars from corpora of language use, and for implementing multi-agent emergent communication experiments that combine symbolic and neural computation. These case studies are meant to demonstrate example usage of the library but are not meant to serve as guidelines or a rule book in any way. As an open-ended framework, PyFCG is envisioned as a tool that empowers its users without harnessing them into a particular way of modeling constructionist approaches to language. We explicitly leave judgment calls on the adequacy of particular theoretical positions or research questions, as well as on the epistemological status of the results that are produced, up to the user of the library.

## FCG in python, really?

Readers who have regularly used FCG might argue that there already exists a stable and mature, efficient, cross-platform, and open-source implementation of FCG, with an active and dedicated, albeit small, developer community.^12^ Indeed, the reference FCG implementation is written in Common Lisp, a dynamic and extensible programming language that, admittedly, excellently fits the project’s requirements, including multi-paradigm, high-level and multi-threaded programming, fast prototyping, and highly efficient symbol processing. So why would anyone spend time and effort on what might look like a Python port?

The reality is that Python has become the most popular programming language in the world^13^ and that, more consequentially, it has also become the dominant language in programming education, as well as today’s *de facto* standard in both linguistics and natural language processing research. Practically speaking, this entails that the success of a software library targeted at the broader computational linguistics community depends before all other things on its compatibility with the Python ecosystem. While this might sound utterly unreasonable, it is not entirely so. For one thing, computer programs typically integrate a variety of external libraries, such as machine learning libraries in the field of computational linguistics, and, for better or for worse, the largest selection of libraries tends to be developed for the most popular programming languages. Another, the investment involved in learning to use a new programming language and environment is a very real obstacle for potential users in today’s time-pressed society.

The development and release of PyFCG follows a trend set by many other libraries that are commonly used in the computational linguistics community. For example, the Stanford CoreNLP Java library^14^ is now accessible from Python through the Stanza library.^15^ Likewise, the PRAAT system for phonetic analysis,^16^ written in C and C++, is now widely used in Python programs via the Parselmouth library.^17^ PyDelphin has been developed as a Python interface to the DELPH-IN suite of tools for processing HPSG grammars.^18^ The Torch library for tensor computation, written in C and Lua, is now even primarily being developed for Python as part of the PyTorch project.^19^

## FCG in python, finally!

Readers who have not yet used FCG, perhaps for the reasons mentioned above, might wonder why it has taken so long for FCG to make its way into the Python ecosystem. It could be ascribed to a lack of actual problems with the existing reference implementation, to the sheer size, scope, and complexity of its codebase, to the difficulty of funding scientific software development, or perhaps most likely, to a combination of all these factors. But fret no more:

$ pip install pyfcg

Once pip-installed, PyFCG can readily be imported as a module into Python programs. It is customary to define fcg as an alias for the PyFCG module so that all functionality is available within the fcg namespace. We initialize PyFCG by calling its init() function, which loads (or downloads if necessary) all external dependencies. PyFCG’s documentation is available on the *Read the Docs* platform^20^ and interactive tutorial notebooks supporting this article can be downloaded from the FCG community website.^21^

>>> import pyfcg as fcg

>>> fcg.init()

On the highest level, PyFCG defines three classes that are of interest to the user: Agent, Grammar, and Construction. The idea is that an agent (of type Agent) has a grammar (of type Grammar), which in turn holds constructions (of type Construction). The Agent class is the main entry point for the user. Upon the creation of a new agent, it is automatically initialized with an empty grammar, that is, a grammar that holds zero constructions.

>>> demo_agent = fcg.Agent()

>>> demo_agent.grammar.size()

0

It was an explicit design choice to tie grammars to agents, emphasizing FCG’s view that a grammar always represents the linguistic knowledge of an individual language user. Grammars are in principle never shared between agents and cannot exist outside an agent. Instances of the Grammar class are therefore only created implicitly via the Agent class.

## PyFCG at work

We present three illustrative case studies that showcase how PyFCG can be used to implement typical use cases of FCG: grammar formalization and testing, learning grammars from semantically annotated corpora, and modeling emergent communication. Each case study is accompanied by an interactive notebook, which can be downloaded from the FCG community website.^21^

### Grammar formalization and testing

A common use of FCG revolves around the formalization and computational operationalization of construction grammar theories and analyses. Not only can computational operationalizations help validate their precision and internal consistency, but they also facilitate the comparison, exchange, and integration of insights from different researchers.^22^ This case study exemplifies how PyFCG can be used to equip an agent with a human-designed grammar, how new constructions can be added to or removed from the agent’s grammar on the fly, how the agent can use its grammar to comprehend and formulate utterances, and how all these processes can be visually inspected through FCG’s graphical web interface. For more information on aspects of the syntax and semantics of FCG that are not particular to the PyFCG module, we refer the interested reader to FCG’s reference documentation.^23^

After importing and initializing PyFCG, we create a new agent named Sue. Sue, as an instance of the fcg.Agent class, is automatically initialized with an empty grammar. Sue is also assigned a unique identifier:

>>> sue = fcg.Agent(name=’Sue’)

>>> sue

<Agent: Sue (id: sue-1) ∼0 cxns>

Sue can load a predefined grammar, specified in the Open FCG Exchange Format (OFEF). OFEF is a JSON-based format for defining FCG constructions and construction inventories across FCG implementations, being at the same time human and machine-readable. We use PyFCG’s load_resource function to download a human-designed demo grammar fragment that specifies seven constructions for processing the English resultative sentence “*Firefighters cut the child free*.”^24^ This grammar fragment uses the Abstract Meaning Representation (AMR)^25^ format to represent constructional meaning, in particular the constructional extension proposed by Bonial et al. (2026).^26^ We instruct Sue to load the grammar specified in the OFEF file by calling their load_grammar_from_file method and then list the names of the constructions that were loaded:

>>> f = fcg.load_resource(’demo-resultative.ofef’)

>>> sue.load_grammar_from_file(f)

>>> sue

<Agent: Sue (id: sue-1) ∼7 cxns>

>>> list(sue.grammar.cxns.keys())

[’firefighters-cxn’, ’child-cxn’, ’cut-cxn’, ’free-cxn’, ’np-cxn’,’resultative-cxn’, ’intransitive-cxn’]

We now ask Sue to comprehend the utterance “*Firefighters cut the child free*.” In FCG, comprehension and formulation respectively refer to the processes of mapping utterances to their meaning representation and vice versa. In order to be able to visually inspect Sue’s comprehension process, we first start up FCG’s graphical web interface and activate the standard trace-fcg monitor. After having comprehended the utterance, we visualize the resulting AMR meaning representation using the more human-readable Penman format:

>>> fcg.start_web_interface()

>>> fcg.activate_monitor(’trace-fcg’)

>>> amr = sue.comprehend(“Firefighters cut the child free.”)

>>> fcg.predicate_network_to_penman(amr)

’(r / resultative-91

 :arg0 (p / person

    :arg0-of (f / fight-01

       :arg1 (f2 / fire)))

 :arg1 (c / child)

 :arg2 (f / free-04

   :arg1 c)

 :arg3 (c2 / cut-01

   :arg0 p))’

We can see that Sue understood that a resultative action took place (denoted by AMR’s resultative-91 roleset), in which the role of “*cause*” (arg0 in resultative-91) is taken up by people who habitually “*fight*” (fight-01) fire (arg1 in fight-01), the role of “*patient*” (arg1 in resultative-91) by a child, the role of “*result*” (arg2 in resultative-91) by the “*unconstrained, unrestricted*” state (free-04) of the child (arg1 in free-04), and the role of “*concomitant event*” (arg3 in resultative-91) by a cutting action in the sense of “*slice, injure*” (denoted by PropBank’s cut-01 roleset^27^) that was performed by an “*intentional cutter*” (arg0 in cut-01), namely the firefighters. A screen capture of the web interface after comprehending the utterance with the more concise trace-comprehension-result monitor activated is shown in Figure 1. The constructional dependency graph^28^ on the left side of the figure shows how seven constructions have combined to process the input utterance, yielding the AMR meaning representation shown on the right.

**Figure 1:**
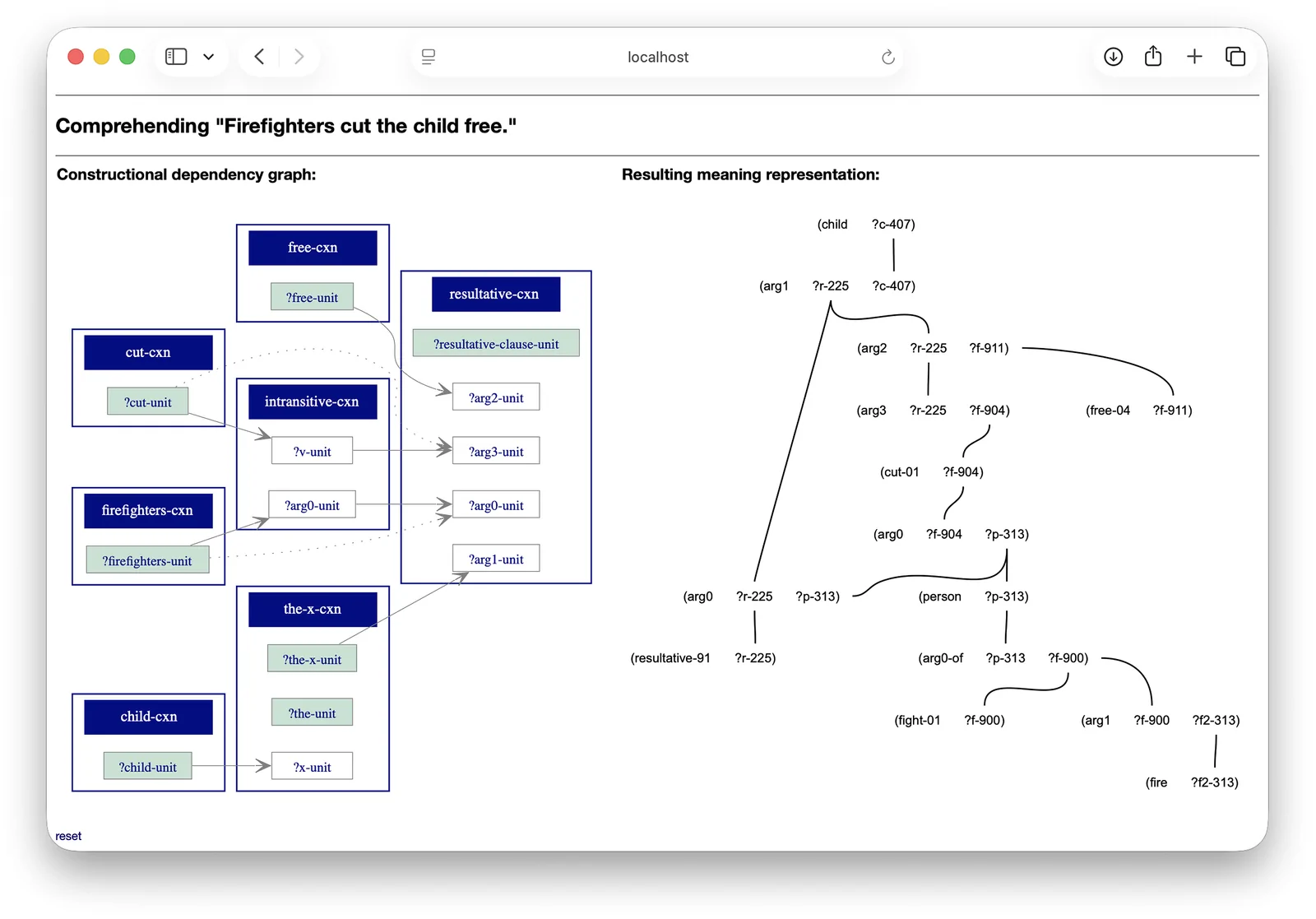
FCG graphical web interface Screenshot of FCG’s web interface after calling sue.comprehend (“Firefighters cut the child free.”) with the trace-comprehension-result monitor activated. The constructional dependency graph on the left shows how seven constructions have combined to yield the AMR representation on the right.

Let us now add a new construction to Sue’s grammar: the dog-cxn that in essence pairs the form “*dog*” with its AMR meaning of instantiating the dog concept. Along with its name, we specify its contributing and conditional poles, and load it into Sue. We also add a categorial link between the category proper to the dog-cxn and the category of the noun slot in the np-cxn, following the design choices made in the demo grammar fragment and very much in the spirit of Radical Construction Grammar.^4^

>>> dog_cxn = fcg.Construction(

name= ’dog-cxn’,

contributing_pole=

[(’?dog-unit’,

{’referent’: ’?d’,

’category’: ’dog-cxn’,

’boundaries’: (’?left’, ’?right’)})],

conditional_pole=

[(’?dog-unit’,

{’#meaning’: [(’dog’, ’?d’)]},

{’#form’: [(’sequence’, ’“dog”’, ’?left’, ’?right’)]})])

>>> sue.add_cxn(dog_cxn)

>>> sue.add_category(’dog-cxn’)

>>> sue.add_link(’dog-cxn’,’np-cxn-n’)

>>> sue

<Agent: Sue (id: sue-1) ∼8 cxns>

We now ask Sue to use their grammar to formulate an utterance that expresses that a cutting action in the sense of “*slice, injure*” was performed by an “*intentional cutter*,” more in particular a person who habitually “*fights*” fire, and that the cutting action itself led to the “*unconstrained, unrestricted*” state of a dog:

>>> amr = ’(r / resultative-91

    :arg0 (p / person

       :arg0-of (f / fight-01

          :arg1 (f2 / fire)))

    :arg1 (d / dog)

    :arg2 (f / free-04

       :arg1 d)

    :arg3 (c / cut-01

       :arg0 p))’

>>> amr = fcg.penman_to_predicate_network(amr)

>>> sue.formulate(amr)

’Firefighters cut the dog free.’

We can see that Sue produces the utterance “*Firefighters cut the dog free.*” Again, the construction application process can be traced in detail in the web interface.

### Learning grammars from corpora

A second use case of FCG concerns the learning of construction grammars from corpora of language use. We take the example of fcg-propbank, an existing FCG subsystem for learning construction grammars from PropBank-annotated corpora.^29^ We demonstrate how a pretrained grammar comprising tens of thousands of constructions can be loaded into an agent and used to extract semantic frames from open-domain text, how a new grammar can be learned from annotated data, and how large grammars can be saved in an efficiently loadable binary format.

As always, we start by creating an agent. In this case, the agent is an instance of the fcg.PropBankAgent class, a subclass of the fcg.Agent class, which is provided by the fcg-propbank subsystem. We download a pretrained, precompiled grammar for English and load it into our agent using its load_grammar_image method. The agent now has at its disposal a grammar that consists of 40,688 constructions.

>>> pb_pretrained = fcg.PropBankAgent()

>>> f = fcg.load_resource(’pb-en.fci’)

>>> pb_pretrained.load_grammar_image(f)

>>> pb_pretrained

<Agent: (id: agent-1) ∼40688 cxns>

Our agent can now use its pretrained grammar to comprehend new utterances. Below, we instruct our agent to comprehend the passive utterance “*Margaret Thatcher was elected Prime Minister of Britain*” (*News on the Web* corpus 19–12–08-us^30^). The resulting meaning representation reveals that the agent identified a single semantic frame that instantiates the elect.01 PropBank roleset (“*elect someone to an office or position*”). The agent also understood the roles of “*candidate*” (arg1) and “*office or position*” (arg2) in this instance of elect.01 are respectively taken up by “*Margaret Thatcher*” and “*Prime Minister of Britain*.”

>>> pb_pretrained.comprehend(“Margaret Thatcher was elected Prime Minister of Britain.”)

[{’roleset’: ’elect.01’,

’roles’: [(’v’,“elected”),

  (’arg1’,“Margaret Thatcher”),

   (’arg2’,“Prime Minister of Britain”)]}]

To enhance human readability, we can again choose to activate an FCG monitor to trace the comprehension process in the web interface. This is illustrated in Figure 2, which shows a visualization of the two frames extracted from the utterance “*She especially enjoyed visiting the old historic churches.*” (*Corpus of Contemporary American English* 1997-spok-cbs_sixty). Please note that this example is meant to demonstrate a typical PyFCG workflow, rather than present a competitive semantic role labeling system. For an extensive discussion of the method and pretrained grammar, we refer the interested reader to Van Eecke and Beuls (2026),^29^ where it is shown that the grammar achieves a frame-level precision of 79.85% with a recall of 80.07% on held-out test sets of the OntoNotes and EWT corpora.

**Figure 2:**
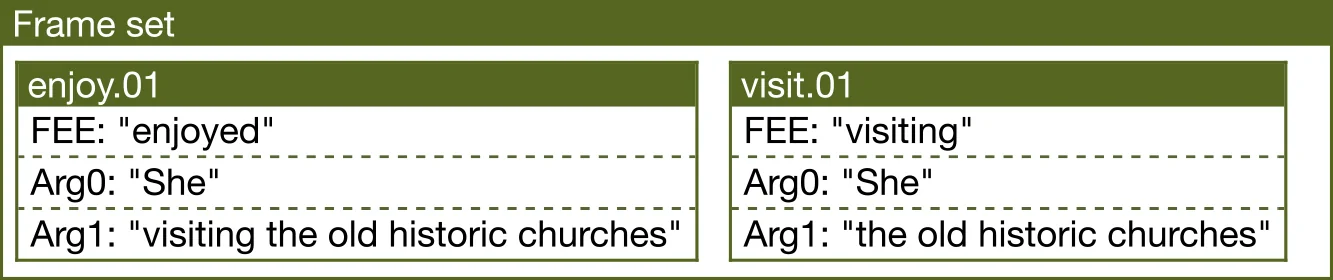
Semantic frames Semantic frames resulting from the comprehension process of the utterance “*She especially enjoyed visiting the old churches.*” (*Corpus of Contemporary American English* 1997-SPOK-CBS_SIXTY).

Let us now create a second agent, again as an instance of the fcg.PropBankAgent class, but let it learn a new grammar from corpus data instead of loading a pretrained one. After having downloaded an example CoNLL file, in which a number of English sentences are annotated with PropBank rolesets, we call the agent’s learn_grammar_from_conll_file method. This call initiates the learning process implemented by the fcg-propbank subsystem and equips the agent with the resulting grammar. In this case, the agent has learned two lexical constructions (for verbs with the lemmas *give* and *send*), two word sense constructions (for the rolesets give.01 and send.01), and two argument structure constructions (a double object construction and a prepositional dative construction).

>>> pb_learner = fcg.PropBankAgent()

>>> f = fcg.load_resource(’pb-annotations.conll’)

>>> pb_learner.learn_grammar_from_conll_file(f)

>>> pb_learner

<Agent: (id: agent-2) ∼6 cxns>

>>> list(pb_learner.grammar.cxns.keys())

[’give(v)-cxn’, ’send(v)-cxn’,

’give.01-cxn’, ’send.01-cxn’,

’arg0(np)+v(v)+arg2(np)+arg1(np)-cxn’,

’arg0(np)+v(v)+arg1(np)+arg2(pp)-cxn’]

We can now instruct our agent to comprehend a previously unseen utterance, using the grammar it just learned, by calling its comprehend method. While comprehending the utterance “*The King of the Belgians sent a box of chocolates to Forrest Gump.*,” the agent identifies an instance of the send.01 (“*give*”) roleset, with “*The King of the Belgians*” as the “*sender*” (arg0), “*a box of chocolates*” as the “*thing sent*” (arg1) and “*to Forrest Gump*” as the “*sent-to*” entity (arg2).

>>> pb_learner.comprehend(“The King of the Belgians sent a box of chocolates to Forrest Gump.”)

[{’roleset’: ’send.01’,

’roles’: [(’v’, “sent”),

   (’arg0’, “The King of the Belgians”),

   (’arg1’, “a box of chocolates”),

   (’arg2’, “to Forrest Gump”)]}]

After learning a grammar, it can be saved by calling the save_grammar_image method of the fcg.Agent class. This method saves the grammar to a file in a compiled, binary format that can later be efficiently reloaded using an agent’s load_grammar_image method.

>>> propbank_agent.save_grammar_image(’usage-based-grammar.store’)

>>> new_agent = fcg.PropBankAgent()

>>> new_agent.load_grammar_image(’usage-based-grammar.store’)

<Agent: (id: agent-3) ∼6 cxns>

Due to licensing restrictions, we are not able to provide large PropBank-annotated corpora as downloadable PyFCG resources. We invite interested readers to obtain such corpora (e.g., OntoNotes or EWT) directly from the *Linguistic Data Consortium*.

### Modeling emergent communication

The final case study exemplifies the primary use case of FCG: implementing the linguistic capability of autonomous agents in agent-based models of emergent communication. In such experiments, agents start out with an empty grammar and gradually build up their linguistic knowledge as they take part in situated communicative interactions with other agents in the population. This case study presents a PyFCG-powered implementation of a neuro-symbolic grounded naming game experiment in which a population of agents converges on a naming convention for referring to entities that they observe in their environment. The difference with earlier versions of the naming game^31^^,^^32^ concerns the representation of entities, which are in this case mugshots of individual people. The choice for a grounded naming game using raw camera images was made to showcase the seamless integration of PyFCG with other widely used Python libraries for subsymbolic processing, in particular PyTorch, NumPy, and scikit-learn, while avoiding the conceptual complexity of experiments on the emergence of more elaborate grammatical structures.

A first step in setting up a grounded naming game experiment concerns the creation of a world in which the agents will operate. In this case, the world consists of 500 mugshots of 10 individual people (396 train images, 104 test images), adopted from the Augmented Olivetti Faces Dataset.^33^^,^^34^ Then, a population of agents is created. We define our agents to be instances of a new class GroundedNGAgent that subclasses from PyFCG’s fcg.Agent class. The agents are thereby initialized with an empty grammar and inherit a collection of methods for interacting with instances of the fcg.Grammar and fcg.Construction classes. We also equip the agents with two neural modules: a randomly initialized embedding matrix that will be used to represent concepts and a small convolutional network designed to take an image and an embedding as input and that will learn to yield a binary output indicating how well a concept fits an image. We use the PyTorch library to straightforwardly implement the neural modules of the agents, as well as for their GPU-accelerated training and deployment.

class GroundedNGAgent(fcg.Agent):

def __init__(self, configuration):

self.concept_embeddings = ConceptNet(...).to(DEVICE)

self.recogniser = VisualRecognitionNet(...).to(DEVICE)

self.recogniser_optimiser = torch.optim.Adam(self.recogniser.

parameters(), lr=1e-3)

self.concept_optimiser = torch.optim.SGD(self.concept_embeddings.

parameters(), lr=0.5)

self.loss_fn = torch.nn.BCELoss()

...

super().__init__()

>>> GroundedNGAgent()

<Agent: agent (id: agent-1) ∼0 cxns>

We also define a new experiment class, GroundedNGExperiment. Its run_series method iteratively calls its run_interaction method, which (i) initiates a new communicative interaction as an instance of the GroundedNGInteraction class, (ii) makes the interaction happen by calling its run method, and (iii) records its outcome.

class GroundedNGExperiment():

...

def run_series(self, nr_interactions, phase=’train’):

for i in range(nr_interactions):

self.run_interaction(phase)

def run_interaction(self, phase=’train’):

# Set phase of experiment

self.phase = phase

# Determine interaction type and create interaction

self.current_interaction = GroundedNGInteraction(self)

# Run actual interaction

self.current_interaction.run(phase=self.phase)

# Record outcome

self.current_interaction.record_outcome()

At the beginning of a new interaction, two agents are drawn from the population and assigned the roles of speaker and listener. At the same time, ten mugshots are sampled from the training set, with the constraint that no individuals are selected more than once. These ten images serve as the scene of the interaction and are made visible to both agents. One image from the scene is randomly selected to be the target of the conversation and is disclosed to the speaker only. The task of the speaker will be to produce an utterance that draws the attention of the listener to the target entity, and the task of the listener will be to point at it.

The speaker first determines whether one of the constructions in its construction inventory discriminates the target from the other entities in the scene. A construction is discriminative for an agent if it fits the target entity better than any other entity in the scene. As agents start the experiment with an empty construction inventory, they will necessarily be unable to find a discriminative construction for the target during their first interactions. If no discriminative construction is found, the speaker agent will create a new construction as a mapping between a randomly generated word form (e.g., “*tezoqiponi*”), an internal embedding index from its own embedding matrix, which is not yet associated with another word (e.g., 0), and an initial entrenchment score (e.g., 0.2). The agent then uses the target image as a positive example and the other images in the scene as negative examples to update the weights of its neural modules (both its CNN and its embedding at index 0). If discriminative constructions were found, the speaker selects the construction with the highest communicative adequacy, defined as the product of its concept-image fit (as yielded by the neural network) and the entrenchment score of the construction. In any case, the word form is then transmitted to the listener agent. The listener agent first calls its comprehend method to map the observed form to an internal embedding index from its own embedding matrix (e.g., 21). It then uses its neural modules (CNN and embedding at index 21) to retrieve the entity in the scene that best fits the given embedding. The listener then points to this entity, after which the speaker provides feedback by pointing at the target image. The agents have achieved communicative success if the listener could correctly identify the target entity. Both agents then shift the weights of their neural modules toward the target entity and away from the other entities in the scene. They also increase the entrenchment score of the successfully used construction, while decreasing the score of competing constructions, defined as constructions that better fit the target but are non-discriminative or have lower communicative adequacy. In case of failure, the listener will decrease the score of its used construction (if applicable), and both agents shift the associated concept embedding toward the target and away from the distractors. If the listener did not have a construction with the observed form, it will create a new construction that maps the observed form to an available internal embedding index with an initial entrenchment score. The listener also updates its neural modules toward the target and away from the distractors.

class GroundedNGInteraction():

...

def run(self, phase=’train’):

“““Defines the interaction script.”””

# Run description task speaker:

self.speaker_process.run(phase)

spoken_utterance = self.speaker_process.utterance

# Run pointing task listener

self.listener_process.run(spoken_utterance)

# Determine interaction success

self.determine_communicative_success()

# Run adoption/reward policy

if phase != ’test’:

self.learn()

A new experiment can be run by creating a new instance of the GroundedNGExperiment class and then calling its run_series methods:

>>> ng = GroundedNGExperiment(...)

>>> ng.run_series(200000, phase=’train’)

200000/200000 [100%] in 2:22:14.0 (23.1/s)

The course of the final communicative interaction of the series, as traced through the FCG web interface, is shown in Figure 3. At the beginning of the interaction, the roles of speaker and listener are respectively assigned to the agents with IDs agent-23 and agent-25. Both agents are placed into a scene consisting of ten mugshots. The 7th mugshot is selected to be the target of the conversation and is disclosed to the speaker. The speaker uses its neural modules to internally conceptualize the target mugshot in terms of the concept embedding stored at index 827, applies its duwurinibu-cxn, and utters the form “*duwurinibu*.” The listener then uses its grammar to map the observed utterance to its own concept embedding at index 811. The listener then uses its neural modules to identify the image in the scene that best fits this concept embedding and points at the 7th mugshot. Finally, the speaker acknowledges that the 7th mugshot was indeed the target of the conversation. Both agents thereby conclude that the communicative interaction was successful and individually reward the constructions that they used while punishing competing ones (not shown in the figure) and updating their neural modules toward the target image and away from the other images in the scene.

**Figure 3:**
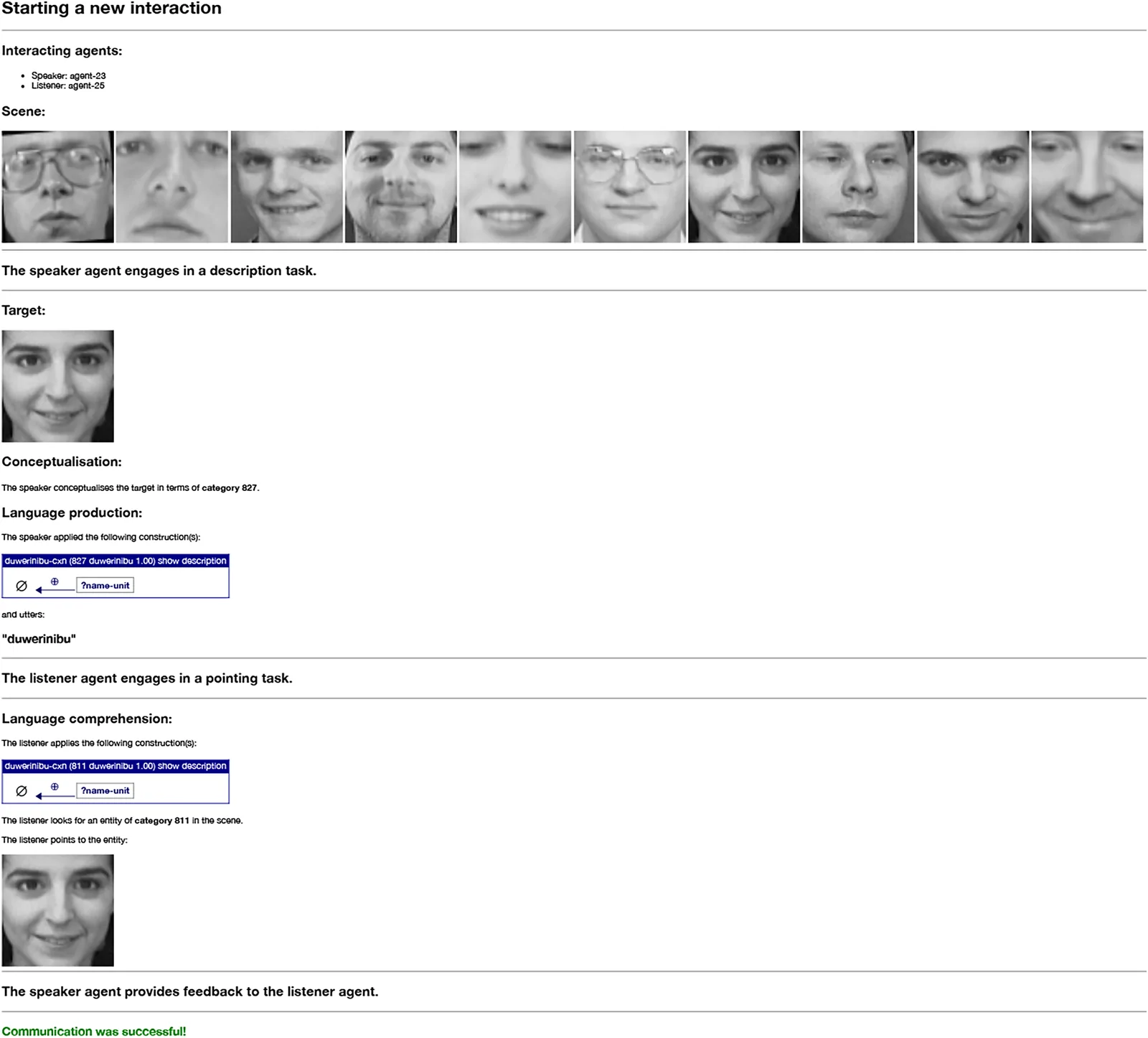
Grounded naming game Graphical trace of a communicative interaction of the grounded naming game experiment, in which agent-23 successfully utters the form “*duwurinibu*” to draw the attention of agent-25 to a previously unseen mugshot. Note that agent-23 and agent-25 internally conceptualize the target as concept embedding 827 and 811, respectively.

The dynamics of an experiment are continuously monitored while running a series of interactions. The outcome of each individual interaction is recorded by the run_interaction method in terms of three metrics.^32^ The first metric quantifies *communicative success* as a binary measure, reflecting whether the listener agent indeed pointed to the target of the conversation. The second metric records the *average number of constructions* in the grammars of the agents. The third metric quantifies the *conventionality* of the uttered form, reflecting whether the listener agent would have uttered the same form under the same circumstances if it had been assigned the speaker role. These recordings can be visualized using any plotting library from Python’s extensive ecosystem, for example, matplotlib, seaborn, or plotly. We demonstrate here the use of matplotlib as implemented by the plot_outcome() method of the GroundedNGExperiment class, which plots the degree of communicative success, degree of conventionality, and average number of constructions as a function of the number of interactions that have taken place:

>>> ng.plot_outcome()

Figure 4 visualizes the dynamics of the training phase of our neuro-symbolic grounded naming game experiment, averaged over 10 independent experimental runs with error bars indicating 2 standard deviations. It shows that the population indeed converges on a communicatively effective naming convention with just above one form for each individual in the world. We can observe the typical language emergence dynamics known from the evolutionary linguistics literature,^35^ where the degrees of communicative success and conventionality gradually increase as more interactions take place, while the average grammar size overshoots in the early stages of the experiment before gradually decreasing as the conventionalization process further unfolds. The graph on top zooms in on the first 200,000 interactions, while the graph at the bottom shows all 1,000,000 training interactions.

**Figure 4:**
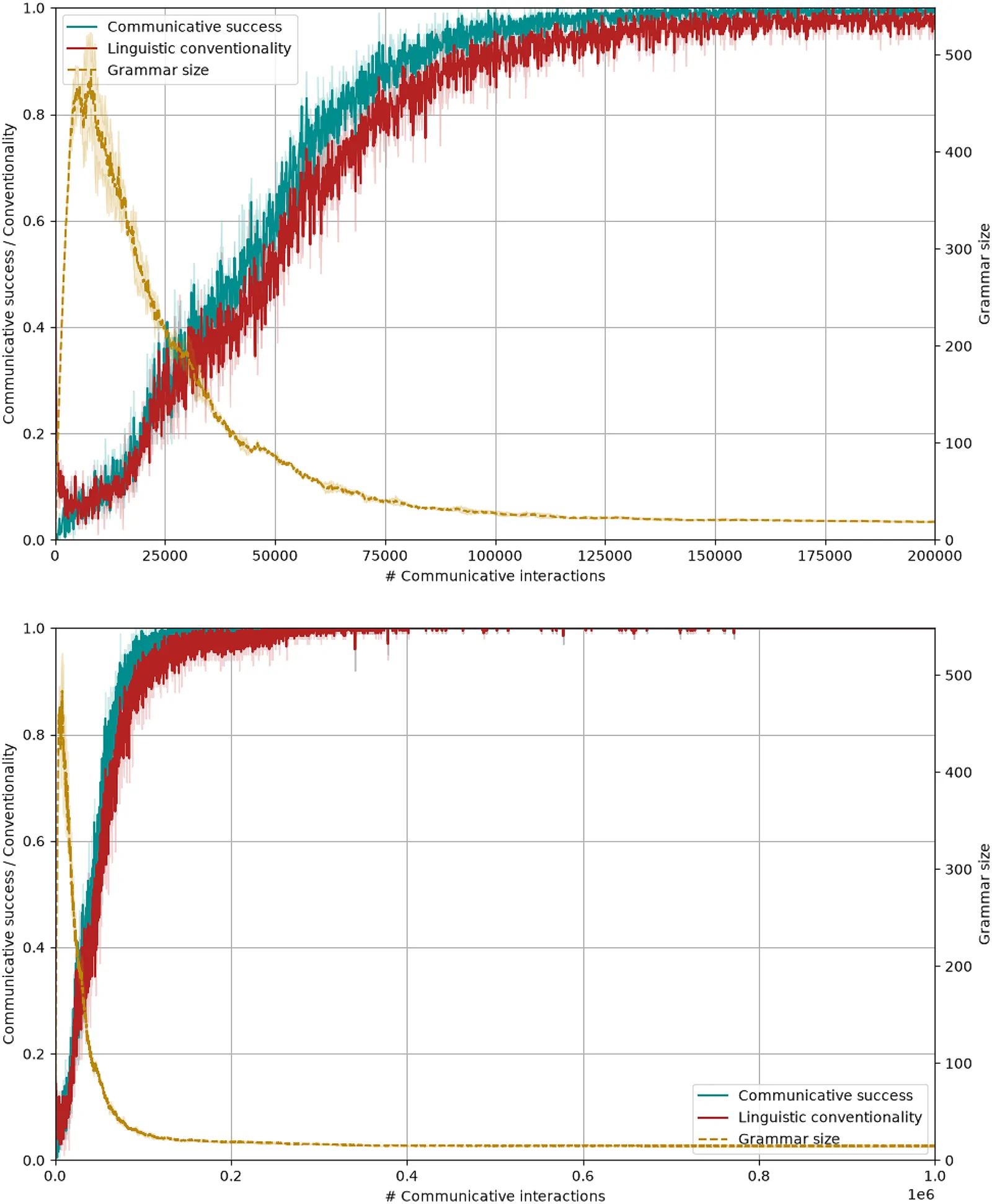
Learning dynamics The dynamics of the PyFCG-powered grounded naming game experiment, with 10 autonomous agents converging on a convention of just over 10 names for referring to the 10 individuals in the world. Mean and two standard deviations over 10 independent runs. The graph on top zooms in on the first 200,000 interactions, while the graph at the bottom shows all 1,000,000 training interactions.

After the training phase has been completed, we can run the experiment on images from the test set, keeping the construction inventory of the agents frozen. For the ten experimental runs reported above, the agents reach a degree of communicative success of 99.45% (± 0.63), a degree of conventionality of 98.98% (± 0.91), and an average of 14.13 used constructions per agent (± 2.28) on the test set.

>>> ng = GroundedNGExperiment(...)

>>> ng.run_series(1000, phase=’test’)

1000/1000 [100%] in 12.6s (79.27/s)

## Technical implementation

The design and technical implementation of PyFCG has been steered by two main considerations. First and foremost, the library needed to feel “native” to Python users, rather than familiar to users already accustomed to the reference FCG implementation. Second, the library needed to wrap the reference implementation, rather than reimplement the original codebase.

A crucial aspect contributing to the “native” look-and-feel of a Python library revolves around the library’s embedding in the Python ecosystem of development tools. PyFCG is implemented as a Python package that is distributed via the Python Package Index (PyPI) and is hence “*pip-installable*” on (at least) macOS, Linux, and Windows. PyFCG’s codebase is versioned using Git and distributed under an open source license, with its documentation being available via the *Read the Docs* platform. Once installed, PyFCG exposes a range of high-level functions, classes, and methods that build on common Python data structures such as objects, dictionaries, lists, and tuples, thereby providing a truly “*Pythonic*” interface to FCG and ensuring maximal compatibility with other Python libraries.

The choice to wrap the reference implementation rather than providing a reimplementation of FCG was motivated by two main reasons. First of all, by wrapping the original Common Lisp implementation, PyFCG can leverage its optimized, multi-threaded codebase and achieve comparable efficiency when running FCG’s most compute-intensive procedures. The second reason was to avoid distributing development and maintenance efforts over two distinct FCG implementations, which could easily become counterproductive in the longer term.

Technically, PyFCG provides a bridge to a stand-alone, executable version of FCG, named *FCG Go*.^36^ Upon calling PyFCG’s init function, FCG Go is downloaded for the correct platform (if necessary) and launched as a background process. PyFCG communicates with this background process through HTTP requests and thereby has access to the complete API of the reference implementation, as well as to its compiled codebase. When instances of PyFCG’s Grammar and Construction classes are created, corresponding objects are instantiated in the FCG Go subprocess. Calls to methods that modify objects of these classes, such as Agent.add_cxn and Construction.set_score, modify their Python representation on the PyFCG side as well as their Common Lisp representation on the FCG Go side. Calls to functions and methods that more generally rely on functionality implemented by the FCG reference implementation or are particularly compute-intensive, such as fcg.start_web_interface and Agent.comprehend are essentially rerouted to the running subprocess. Finally, functions and methods that do not modify FCG objects and are not compute-intensive, including those for listing an agent’s constructions, for retrieving the scores of constructions, and for inspecting their internal representations, only involve objects on the Python side.

Crucially, the architecture of PyFCG ensures that its users do not notice that the library interfaces with a non-Python subprocess. The subprocess is automatically launched when the library is initiated, without the need to install a Common Lisp environment or any other dependencies. PyFCG’s public interface is defined in terms of Pythonic data structures; any errors that would arise in the Common Lisp subprocess are caught and transposed to Python Exceptions (subclass FcgError), and the subprocess itself is safely closed by a clean-up method when Python exits. Moreover, as the code run by the FCG Go subprocess is exactly the same compiled code that would be run from the reference FCG distribution, the only computational overhead that is involved when comparing to the reference implementation is the cost of an HTTP round trip measured at about 5 ms on an Apple M4 Max MacBook Pro. For reference, a call to comprehend(-all) or formulate(-all) only takes a single HTTP round trip.

## Discussion and conclusion

This article has presented PyFCG as an open-source software library that brings FCG to the Python programming language. PyFCG is publicly distributed as a pip-installable Python package and provides a fully Pythonic interface to FCG’s reference implementation. The library thereby enables its users to seamlessly integrate constructional language processing and learning into Python programs and to combine this functionality with that of other libraries within Python’s extensive ecosystem. At the same time, the design of PyFCG as a wrapper around an executable version of FCG’s Common Lisp reference implementation, which stealthily runs its multi-threaded and compute-intensive procedures in the background, ensures that PyFCG can incorporate all FCG functionality and that the community’s development and maintenance efforts can remain united.

Apart from a general description of the library, its motivation, and its technical implementation, this article has presented three illustrative case studies that showcase how PyFCG can be integrated into typical use cases of FCG. The first case study has demonstrated how FCG agents can be created, how they can be equipped with a human-designed grammar, how they can be instructed to comprehend and formulate natural language utterances, and how these processes can be visually inspected using FCG’s web interface. The second case study has showcased how FCG agents can learn grammars from semantically annotated corpora, how they can use these grammars to annotate new data, and how grammars consisting of tens of thousands of constructions can efficiently be saved and later reloaded into agents. The final case study has demonstrated the use of PyFCG in setting up agent-based experiments on emergent communication. We have taken the example of a grounded naming game experiment and have shown how the seamless integration of high-level FCG functionality with Python’s PyTorch library for implementing neural networks can ease the implementation of the capacities of individual agents to learn language from communicative interactions situated in grounded scenarios.

FCG is being developed as an open-source community project, which brings together the construction grammar and computational linguistics communities. While strong ties between both communities have existed since the foundation of the field of construction grammar in the 1980s, recent initiatives such as UCxn^37^ and the Construction Grammars and NLP (CxGs+NLP) workshop series,^38^^,^^39^ along with an increasing volume of work on the relationship between constructions and Large Language Models,^40^^,^^41^^,^^42^^,^^43^^,^^44^^,^^45^^,^^46^^,^^47^ bear witness to a growing interest in research at the intersection of both fields. By bringing FCG into the Python ecosystem, we hope to create new opportunities for researchers to further integrate constructional language processing with neural network architectures. While this goal is not new,^48^^,^^49^^,^^50^ PyFCG has the potential to make its computational operationalization significantly more straightforward.

As we reach the end of this article, it is useful to remind ourselves that FCG was conceived as an open instrument that provides a collection of high-level building blocks for constructional language processing, with the goal of supporting research and applications at the intersection of construction grammar and computational linguistics. The practical case studies described in this article, along with their accompanying interactive notebooks, demonstrate example usage of the library but are not meant to serve as guidelines or rule books in any way. FCG has always evolved to fit the needs of its users, and new perspectives or use cases brought by PyFCG users or contributors will be heartily embraced.

## Acknowledgments

We are grateful to Remi van Trijp for his insightful comments on an earlier version of this manuscript as well as the two anonymous iScience reviewers. The FCG Go logo was designed by Liesbet De Vos and the icons in the graphical abstract are from Flaticon.com. This research was supported by the F.R.S.-10.13039/501100002661FNRS-10.13039/501100003130FWO WEAVE project HERMES I under grant numbers T002724F (F.R.S.-FNRS) and G0AGU24N (FWO), the Flemish Government under the Onderzoeksprogramma Artificiële Intelligentie (AI) Vlaanderen program and the AI Flagship project ARIAC by DigitalWallonia4.ai.

## Author contributions

Conceptualization: P.V.E. and K.B; Software: P.V.E. and K.B; Visualization: P.V.E. and K.B.; Writing – original draft and revisions: P.V.E. and K.B. Both authors approved the final submitted manuscript.

## Declaration of interests

The authors declare no competing interests.

## Declaration of generative AI and AI-assisted technologies in the manuscript preparation process

The authors declare that this article was conceived and written without the use of generative AI tools.
